# Supplementary material for: Hundred-fold increase in SARS-CoV-2 spike antibody levels over three years in a hospital clinical laboratory
Source: Microbiol Spectr. 2023 Oct 9;11(6):e02183-23. doi: 10.1128/spectrum.02183-23 (PMC10715067; doi:10.1128/spectrum.02183-23)
Supplement: Supplemental material — Tables S1 to S3 and Fig. S1. [file spectrum.02183-23-s0001.docx]

**Supplemental Tables and Figures**

**Table S1**. Key demographic characteristics (gender and age) and spike IgG levels by time period. IQR= interquartile range. At each time period, mean age was compared in the two genders by simple linear regression, and median spike IgG levels by Wilcoxon ranksum test.

| **Time** | **Females (No. = 6,362)** | | | **Males (No. = 4,660)** | | |  |  |
| --- | --- | --- | --- | --- | --- | --- | --- | --- |
|  | *No.* | *Age*  *(x ± SD)* | *Spike IgG*  *(median and IQR)* | *No.* | *Age*  *(x ± SD)* | *Spike IgG*  *(median and IQR)* | *p value*  *for age* | *p value*  *for IgG* |
| Apr - Dec 2020 | 1519 | 48 (±20) | 0.12 (0.3) | 1371 | 47 (±22) | 0.14 (0.4) | 0.760 | 0.002 |
| Jan - Jun 2021 | 1441 | 51 (±21) | 2.7 (7.6) | 1172 | 51 (±22) | 1.2 (6.6) | 0.798 | <0.001 |
| Jul - Nov 2021 | 1854 | 52 (±18) | 5.4 (7.2) | 959 | 53 (±21) | 4.7 (7.9) | 0.255 | 0.009 |
| Dec 21 - Jun 22 | 1039 | 50 (±20) | 12 (48) | 748 | 52 (±23) | 11 (46) | 0.083 | 0.068 |
| Jul 22 - Feb 23 | 509 | 49 (±20) | 51 (109) | 410 | 48 (±23) | 44 (83) | 0.588 | <0.001 |

**Table S2**. Median, minimum, and maximum spike IgG levels by time period. Results are shown for both the entire dataset of 15,820 sera and for the subset of 10,271 that tested positive (that is, had a value greater than 1.23 optical density ratio). P values were calculated by multiple linear regression to assess the mean log_10_ of the spike IgG levels across the five time periods, adjusting for gender.

| **Time** | **All sera (No. = 15,820)** | | | | **Positive sera (no. = 10,271)** | | | |  |
| --- | --- | --- | --- | --- | --- | --- | --- | --- | --- |
|  | *No.* | *Median* | *Minim.* | *Maxim.* | *No.* | *Median* | *Minim.* | *Maxim.* | *P vs. previous period* |
| Apr - Dec 2020 | 3109 | 0.13 | .01 | 154 | 460 | 6.4 | 1.25 | 154 | - |
| Jan - Jun 2021 | 3039 | 1.0 | .01 | 294 | 1620 | 7.1 | 1.24 | 294 | <0.0001 |
| Jul - Nov 2021 | 3724 | 5.3 | .01 | 787 | 2807 | 6.9 | 1.24 | 787 | <0.0001 |
| Dec 21 - Jun 22 | 3526 | 11.7 | .01 | 2272 | 3078 | 17.6 | 1.26 | 2272 | <0.0001 |
| Jul 22 - Feb 23 | 2422 | 48.7 | .01 | 2851 | 2306 | 52.1 | 1.24 | 2851 | <0.0001 |

**Table S3**. Maryland data on COVID-19 reported cases, percent vaccinated, percent positive for spike antibodies in SeroNet (seroprevalence), and percent positive blood donors. Maryland has an estimated population size of 6.1 million as of 2023. *Cumulative cases were obtained at- <https://opendata.maryland.gov/Health-and-Human-Services/MD-COVID-19-Total-Cases-Statewide/t7ek-pn7n>. Maryland cumulative vaccine history at <https://covid.cdc.gov/covid-data-tracker/#vaccination-trends>. ***COVID-19 seroprevalence from CDC Seronet <https://covid.cdc.gov/covid-data-tracker/#national-lab> through February 17, 2022. ****blood donor seroprevalence estimates from <https://covid.cdc.gov/covid-data-tracker/#nationwide-blood-donor-seroprevalence-2022> through December 2022.

| *Study period* | *End date* | *Cumulative COVID-19 cases** | *Received at least one dose (%)*** | *Completed primary series (%)*** | *Received updated bivalent dose (%)*** | *Sero-*  *Prevalence*  *(SeroNet,)(%)**** | *Blood donors (%)***** | *Sero-*  *Prevalence*  *(this study)* |
| --- | --- | --- | --- | --- | --- | --- | --- | --- |
| Apr - Dec 2020 | 12-31-2020 | 276,662 | 1.4 | 0 | 0 | 15 | 7 | 15 |
| Jan - Jun 2021 | 06-30-2021 | 462,279 | 58 | 64 | 0 | 27 | 94 | 53 |
| Jul - Nov 2021 | 11-30-2021 | 586,143 | 79 | 70 | 0 | 26 | 96 | 75 |
| Dec 21 - Jun 22 | 06-30-2022 | 1,132805 | 88 | 77 | 0 | 50 | 94 | 87 |
| Jul 22 - Feb 23 | 02-28-2023 | 1,356,871 | 92 | 80 | 23 | no data | 98 | 95 |

**Fig. S1** Monthly means over the time periods indicating a plateau in the rise in antibody levels after April 2022, month 24 of the study. Means and 95% upper and lower limits of the mean are plotted for each month. The conversion of Euroimmun AU 1=14 RU/mL=45 BAU/mL.
